# Prevalence, genomic characterization and antimicrobial resistance of *Campylobacter* spp. isolates in pets in Shenzhen, China

**DOI:** 10.3389/fmicb.2023.1152719

**Published:** 2023-06-01

**Authors:** Changyan Ju, Yanping Ma, Bi Zhang, Guilan Zhou, Hairui Wang, Muhua Yu, Jiaoming He, Yongxiang Duan, Maojun Zhang

**Affiliations:** ^1^Laboratory, Nanshan Center for Disease Control and Prevention, Shenzhen, China; ^2^Clinic, IVC Shenzhen Animal Hospital, Shenzhen, China; ^3^State Key Laboratory of Infectious Disease Prevention and Control, Chinese Center for Disease Control and Prevention, Beijing, China

**Keywords:** *Campylobacter upsaliensis*, *Campylobacter jejuni*, *Campylobacter helveticus*, whole-genome sequencing, antimicrobial resistance, matrix-assisted laser desorption ionization-time of flight mass spectrum, pets, virulence-associated genes

## Abstract

The prevalence of *Campylobacter spp*.in pets is a potential concern for human health. However, little is known about the pet-related *Campylobacter* spp. in China. A total of 325 fecal samples were collected from dogs, cats, and pet foxes. *Campylobacter* spp. were isolated by culture, and MALDI-TOF MS was used to identify 110 *Campylobacter* spp. isolates in total. *C. upsaliensis* (30.2%, 98/325), *C. helveticus* (2.5%, 8/325), and *C. jejuni* (1.2%, 4/325) were the three found species. In dogs and cats, the prevalence of *Campylobacter* spp. was 35.0% and 30.1%, respectively. A panel of 11 antimicrobials was used to evaluate the antimicrobial susceptibility by the agar dilution method. Among *C. upsaliensis* isolates, ciprofloxacin had the highest rate of resistance (94.9%), followed by nalidixic acid (77.6%) and streptomycin (60.2%). Multidrug resistance (MDR) was found in 55.1% (54/98) of the *C. upsaliensis* isolates. Moreover, 100 isolates, including 88 *C. upsaliensis*, 8 *C. helveticus*, and 4 *C. jejuni*, had their whole genomes sequenced. By blasting the sequence against the VFDB database, virulence factors were identified. In total, 100% of *C. upsaliensis* isolates carried the *cadF, porA, pebA, cdtA, cdtB*, and *cdtC* genes. The *flaA* gene was present in only 13.6% (12/88) of the isolates, while the *flaB* gene was absent. By analyzing the sequence against the CARD database, we found that 89.8% (79/88) of *C. upsaliensis* isolates had antibiotic target alteration in the *gyrA* gene conferring resistance to fluoroquinolone, 36.4% (32/88) had the aminoglycoside resistance gene, and 19.3% (17/88) had the tetracycline resistance gene. The phylogenetic analysis using the K-mer tree method obtained two major clades among the *C. upsaliensis* isolates. All eight isolates in subclade 1 possessed the *gyrA* gene mutation, the aminoglycoside and tetracycline resistance genes, and were phenotypically resistant to six classes of antimicrobials. It has been established that pets are a significant source of *Campylobacter* spp. strains and a reservoir for them. This study is the first to have documented the presence of *Campylobacter* spp. in pets in Shenzhen, China. In this study, *C. upsaliensis* of subclade 1 required additional attention due to its broad MDR phenotype and relatively high *flaA* gene prevalence.

## 1. Introduction

Campylobacteriosis is one of the most common bacterial foodborne illnesses worldwide (Campagnolo et al., 2018; Hudson et al., 2021). Although in most cases, campylobacteriosis is manifested as mild and self-limiting, in a small percentage of people, *Campylobacter* infection is a precursor of more serious illnesses, including Guillain–Barré syndrome (GBS) and Miller–Fisher Syndrome (MFS) (Moore et al., 2005; Finsterer, 2022; Latov, 2022).

Between 2012 and 2015, FoodNet identified 39,345 culture-confirmed *Campylobacter* infections; *C. jejuni* and *C. coli* were the main identified species in the USA (Patrick et al., 2018). Although *C. upsaliensis* is significantly less pathogenic than *C. jejuni*, it cannot be considered non-pathogenic (Bojanić et al., 2020). *C. upsaliensis* has been isolated from human blood, placental tissue, breast abscess, and stool (Couturier et al., 2012), and it was also isolated from an infected large hepatic cyst (Ohkoshi et al., 2020). Moreover, Nakamura *et al*. described a severe fatal infection caused by *C. upsaliensis* that killed a 70-year-old woman (Nakamura et al., 2015).

A known risk factor for human campylobacteriosis is contact with dogs and cats (Acke, 2018; Martinez-Anton et al., 2018). Ownership greatly raised the risk of pet-associated human *C. jejuni* or *C. coli* infection (Mughini et al., 2013; Campagnolo et al., 2018). For companion animals, *C. upsaliensis, C. jejuni*, and *C. helveticus* in dogs and *C. helveticus, C. upsaliensis*, and *C. jejuni* in cats are the *Campylobacter* spp. most frequently isolated from fecal samples (Acke, 2018). Flagellum, several flagellum-secreted components, protein adhesins, cytolethal distending toxin (CDT), lipooligosaccharide (LOS), and serine protease HtrA are the virulence-associated bacterial determinants in *Campylobacter* spp. (Tegtmeyer et al., 2021). These virulence factors may harm the intestine directly through cell invasion or toxin generation or indirectly through other means (Lopes et al., 2021). Analysis of the virulence factor is critical for improved diagnosis, surveillance, and control (Bolton, 2015).

A growing issue is that *Campylobacter* species are becoming more antibiotic-resistant, making therapy more challenging. In total, 83.1% of the *Campylobacter* spp. isolates from chickens and pigs were multidrug-resistant (MDR), and 99% were resistant to at least one antimicrobial agent (Choi et al., 2021). Nalidixic acid, ciprofloxacin, tetracycline, ampicillin, azithromycin, chloramphenicol, and gentamicin were typically the antimicrobials with the highest rate of resistance (Shakir et al., 2021; Asuming-Bediako et al., 2022; Behailu et al., 2022; Ščerbová et al., 2022).

Whole-genome sequencing (WGS) has become more widely used due to falling costs. WGS is the most informative approach for characterizing bacterial isolates (Llarena et al., 2017; Cantero et al., 2018; Rokney et al., 2020). It can provide a variety of information, including identifying genes encoding for antimicrobial resistance, detecting virulence factors, subtyping, and the source contribution to outbreaks (Bravo et al., 2021; Prendergast et al., 2022). Moreover, the plasmid-borne type VI secretion system in *C. coli* was discovered by WGS (Ghatak et al., 2017). Additionally, *C. lari* strain SCHS02 was shown to be genetically related to human clinical isolates and isolates from bird samples rather than isolates from other environmental sources (Song et al., 2020).

This study evaluated the prevalence and genetic characteristics of *Campylobacter* spp. in pets to assess the possibility of human infection with *Campylobacter* from pets in Shenzhen, China.

## 2. Materials and methods

The institutional review board of the Nanshan Center for Disease Control and Prevention approved the study.

### 2.1. Culture, isolation, and identification

A total of 325 feces samples from dogs (*n* = 217), cats (*n* = 103), and pet foxes (*n* = 5) were taken from 16 chain animal hospitals in Shenzhen, China, between September and November 2019. From the pet owners' responses to surveys, we gathered informed permission and data on the animal's age, sex, weight, clinical signs, and past usage of antimicrobials. We sorted the animals into groups based on their age, weight, and medical conditions and then compared the prevalence of *Campylobacter* in each group. SPSS26.0 (IBM Corp., Armonk, NY, United States) was used for statistical analysis, and the count data were compared between the groups using the chi-square test (χ^2^). The statistical significance of the difference was indicated by a statistical probability of <0.05 (*P* < 0.05).

The feces samples (~5 g) were taken in a 5 ml threaded pipe with sampling solution (brain heart infusion medium with 20% glycerin) and transferred to the laboratory at 4°C within 4 h for bacterial isolation. We isolated *Campylobacter* using *Campylobacter* isolation commercial kits (ZC-CAMPY-001, Qingdao Sinova Bio-technology Co. Ltd, Qingdao, China). In brief, we vortexed the sampling solution and inoculated it into 4 ml enrichment broth with approximately 0.5 ml of the solution. The main component of the enrichment broth was the modified Preston broth with vancomycin, trimethoprim, and amphotericin B. The broth was then incubated at 37°C for 24 h in a microaerobic atmosphere (5% O_2_, 10% CO_2_, and 85% N_2_), provided by Anoxomat Mark II, the Netherlands. Approximately 300 μl of the enrichment medium was spotted on the surface of a 0.45 μm cellulose membrane filter from the kit, which was pasted onto Karmali and Columbia agar plates. After being incubated for 24 h at 37°C in a microaerophilic environment, at least four small round and whitish colonies of 2 mm in diameter were streaked on blood agar plates to obtain the pure culture. Matrix-assisted laser desorption/ionization time-of-flight mass spectrometry (MALDI-TOF MS), as previously described (Hsieh et al., 2018; Lawton et al., 2018), was used to identify suspicious colonies using the RUO Bacterial Test Standard (bioMérieux).

### 2.2. Antimicrobial susceptibility testing

All *Campylobacter* isolates' minimum inhibitory concentration (MIC) was calculated and performed using commercial kits from Qingdao Sinova Bio-technology Co. Ltd, Qingdao, China. The agar dilution method was used, and the breakpoints used for 11 antimicrobials were as follows:

I. The Clinical and Laboratory Standards Institute (CLSI) M45 (3rd Edition, 2015) for *C. jejuni* was comprised as follows:

1. erythromycin (≥ 32 μg ml^−1^)
2. ciprofloxacin (≥ 4μg ml^−1^)
3. tetracycline (≥ 16μg ml^−1^).

II. The CLSI M100-M02-M07 (2015) for Enterobacteriaceae was included:

1. chloromycetin (≥ 32 μg ml^−1^)
2. gentamycin (≥ 16 μg ml^−1^)
3. azithromycin (≥ 32 μg ml^−1^)
4. nalidixic acid (≥ 32 μg ml^−1^)
5. streptomycin: there are no MIC interpretive standards.

III. Combined with NARMS 2015 for *C. jejuni* and consisting of

1. telithromycin (≥ 8μg ml^−1^)
2. florfenicol (≥ 8μg ml^−1^)
3. clindamycin (≥ 1μg ml^−1^).

IV. EUCAST for *C. jejuni*

streptomycin (≥ 4 μg ml^−1^).

In brief, to create a suspension of 0.5 McFarland turbidity, *Campylobacter* colonies were suspended in NaCl solution. Colony-forming units (CFU) per ml were determined by diluting 100 μl of the suspension in 900 μl of 0.9% NaCl solution. We employed a 96-well microplate covered with Mueller–Hinton agar, sheep blood, and 11 antimicrobials. Each well was dispensed with 2 μl of the suspension. The plates were incubated at 37°C for 48 h in a microaerophilic atmosphere. The MIC was defined as the lowest concentration of each antimicrobial that could prevent visible growth, and *C. jejuni* ATCC33560 was used as the control. In addition, this study defined multidrug resistance (MDR) as resistance to three or more classes of antimicrobials.

### 2.3. Whole-genome sequencing (WGS)

One or two blood agar plates (Huankai Biology, Guangzhou, China) of *Campylobacter* were needed to obtain sufficient material for DNA extraction. The colonies were resuspended in tubes containing 1 ml of phosphate-buffered saline (PBS). The tubes were centrifuged at 8,000 rpm for 15 min before removing the supernatant. Following the manufacturer's instructions, the resultant pellet was further processed for DNA recovery using the commercial nucleic acid extraction kit (Tianlong, China). The double-stranded DNA (dsDNA) concentration was examined with a microplate spectrophotometer (Epoch, BioTek, USA). Novo Source Technology Co., Ltd. (Beijing, China) used the NovaSeq PE150 (Illumina, San Diego, CA, USA) to perform WGS when the DNA mass was more than 1 μg.

After sequencing, the WGS raw data were trimmed and *de novo* assembled by CLC Genomics workbench 12 (QIAGEN Bioinformatics). In brief, the sample DNA was randomly interrupted to construct a DNA library for double-end sequencing. Quality trimming was based on quality scores with a limit cutoff of 0.05 and an ambiguity number of ≤ 2. Filter options were the minimum frequency of 2.0%, the minimum forward/reverse balance of 0.05, and the minimum average base quality of 20.0. Low-quality reads were removed if the quality scores of ≥ 3 consecutive bases were ≤ Q30, and then, the high-quality reads were assembled. The assembly parameters were the automatic word size of 45, the automatic bubble size of 98, and the minimum contig length of 500.

To identify the species, the sequence was blasted against the *Campylobacter* database downloaded from PubMed (2021-11-10). Next, the ResFinder database (2021-11-20) and Comprehensive Antibiotic Resistance Database (CARD, 2023-2-22) were used to acquire the antimicrobial resistance genes. Finally, the results from CARD were used. The setting options in CARD were “perfect and strict hits only”, “exclude nudge”, and “high quality/coverage”. We used the ≥ 87% identity of the matching region as the cutoff value. BLASTn was used to find a ciprofloxacin resistance-causing nucleotide mutation in the *gyrA* gene. The presence of virulence factors was determined by submitting the assembled genomes to the virulence factor database (VFDB, 2021-12-15) (http://www.mgc.ac.cn/cgi-bin/VFs/v5/main.cgi?func=VFanalyzer), and R language (4.1.2) was used to obtain the heatmap of a virulence factor of *C. upsaliensis*. Phylogenetic analysis was conducted by the K-mer tree method with the CLC Genomics workbench 12. K-mers are substrings of length K contained within a biological sequence. The K-mer length in this study was 16, the only index k-mers with the prefix ATGAC, and feature frequency profile (FFP) *via* Jensen–Shannon divergences was used to construct a phylogenetic tree by the neighbor-joining algorithm, which is a distance-based method that can create trees based on multiple single sequences. Reference strains were downloaded from PubMed, and a sequence length of > 1.5Mb was selected. Finally, we annotated the phylogenetic tree by the ITOL website (v6).

## 3. Results

### 3.1. Prevalence of *Campylobacter* spp. from pets

A total of 110 *Campylobacter* spp. isolates were identified by MALDI-TOF MS. Among the pets with *Campylobacter* spp., 99.1% (108/109) harbored a single type of *Campylobacter*, and only one dog (0.1%, 1/109) carried *C. upsaliensis* and *C. jejuni* concurrently. The overall prevalence of *Campylobacter* spp. in pets was 33.5% (109/325). *C. upsaliensis* (30.2%, 98/325) showed the highest prevalence, followed by *C. helveticus* (2.5%, 8/325) and *C. jejuni* (1.2%, 4/325). According to Table 1, the proportion of dogs and cats with *Campylobacter* spp. was 35.0% and 30.1%, respectively.

**Table 1:** The prevalence of *Campylobacter* spp. from pets' feces samples in Shenzhen, China.

**Sources**	**Clinical symptom**	***C. upsaliensis***	***C.jejuni***	***C.helveticus***	**Total prevalence rate**
Cats (*n =* 103)	Diarrhea	16.7% (5/30)	1.3% (1/30)	0 (0/30)	30.1% (31/103)
		Non-diarrhea	23.3% (17/73)	2.7% (2/73)		8.2% (6/73)	
Dogs (*n =* 217)	Diarrhea	22.2% (10/45)	0 (0/45)	0 (0/45)	35.0% (76/217)
		Non-diarrhea	36.6% (63/172)	0.6% (1/172)		1.2% (2/172)	
Pet foxes (5)	Diarrhea	0 (0/2)	0 (0/2)	0 (0/2)	60% (3/5)
		Non-diarrhea	100% (3/3)	0 (0/3)		0 (0/3)	

From the questionnaire, 20.7% (45/217) of dogs exhibited diarrheal symptoms, whereas 79.3% (172/217) did not. 51.6% (112/217) of dogs weighed over 5 kg, and 48.4% (105/217) of dogs weighed <5 kg. 67.7% (147/217) of the dogs were older than 3 years old compared to 32.3% (70/217) of dogs younger than 3 years old. For cats, there were symptoms of diarrhea in 29.1% (30/103) and none in 70.8% (70/103). 51.6% (75/103) weighed over 2 kg, whereas 48.4% (28/103) were under 2 kg. 9.2% (96/103) of the cats were younger than 3 years old, and 6.8% (7/103) were older than 3 years old. Three *C. jejuni* strains were found in two cats and one dog without diarrhea, and one *C. jejuni* strain was found in a cat with diarrhea.

Antimicrobials were used by 24.6% (80/325) of the pets in the past 6 months, and the most frequently used antimicrobials were beta-lactams (58.8%, 47/80).

There was no statistically significant difference in the prevalence of *Campylobacter* spp. between the diarrhea group and the non-diarrhea group among dogs or cats. No statistically significant difference was identified between dogs or cats that were younger (3 years old) or older (> 3 years old). Moreover, there was no statistically significant difference in the prevalence among the pets' weights.

### 3.2. Antimicrobial susceptibility

The antimicrobial susceptibility of all 110 isolates was determined by the agar dilution method, and the test was repeated twice in parallel. We present the results in Supplementary Table 1.

The MICs and the percentage of resistant isolates of *C. upsaliensis* are shown in Table 2. Ciprofloxacin had the highest rate of resistance (94.9%), followed by nalidixic acid (77.6%) and streptomycin (60.2%). 84.7% (83/98) of the *C. upsaliensis* isolates were resistant to at least two classes of antimicrobials. Multidrug resistance (MDR, resistant to at least three classes of antimicrobials) was found in 55.1% (54/98) of the isolates. The main MDR pattern (35.7%, 35/98) was aminoglycosides/quinolones/lincosamides. Furthermore, 12 isolates were resistant to six classes of antimicrobials, namely aminoglycosides,/ketolides,/macrolides,/quinolones,/lincosamides, and/tetracyclines.

**Table 2:** MICs and resistance of 98 isolates of *C. upsaliensis* to the antimicrobials.

**Antimicrobial class**	**Antimicrobial agent**			**Percentage of all isolates with MIC(**μ**g mL**^**−1**^**)**							**Total resistance rate (%)**
		≤ **0.25**	≤ 0.50^*^	**1**	**2**	**4**	**8**	**16**	≥32^**^	≥**64**	
Aminoglycosides	Gentamicin		34.7	15.3	2.0	2.0	2.0	3.1^#^	1.0	39.8	43.9
	Streptomycin		1.0	15.3	23.5	3.1	4.1	4.1	3.1	45.9	60.2
Ketolide	Telithromycin	8.2	5.1	15.3	25.5	12.2	13.3	1.0	19.4		33.7
Macrolides	Azithromycin		61.2	11.2	2.0	1.0	1.0	0.0	0.0	23.5	23.5
	Erythromycin		10.2	15.3	29.6	16.3	5.1	0.0	0.0	23.5	23.5
Quinolones	Ciprofloxacin		0.0	0.0	5.1	7.1	10.2	19.4	38.8	19.4	94.9
	Nalidixic acid		0.0	1.0	1.0	3.1	4.1	13.3	12.2	65.3	77.6
Lincosamides	Clindamycin	25.5	20.4	20.4	6.1	4.1	7.1	4.1	12.2		54.1
Phenicols	Florfenicol		5.1	17.3	26.5	34.7	12.2	4.1	0.0	0.0	16.3
	Chloromycetin		1.0	9.2	21.4	28.6	27.6	12.2	0.0	0.0	0.0
Tetracyclines	Tetracycline		11.2	16.3	20.4	22.4	8.2	2.0	1.0	18.4	21.4

^*^This column was equal to 0.5 g ml^−1^ for telithromycin and clindamycin and ≤ 0.50 g ml^−1^ for other antimicrobials.

^**^This column was ≥32 g ml^−1^ for telithromycin and clindamycin and equal to 32 g ml^−1^ for other antimicrobials.

^#^Numbers in the shaded area indicate the percentages of resistant isolates defined by the breakpoint.

All four *C. jejuni* isolates were MDR. The susceptible antimicrobials were erythromycin, azithromycin, gentamycin, and telithromycin.

One of the eight *C. helveticus* isolates was MDR, macrolides/quinolones/aminoglycosides/phenicols. All eight isolates were susceptible to telithromycin, clindamycin, tetracycline, and chloromycetin. Three *C. helveticus* isolates were susceptible to all the antimicrobials in this study.

### 3.3. Whole-Genome sequencing and virulence factor

A total of 100 isolates including 88 *C. upsaliensis*, 8 *C. helveticus*, and 4 *C. jejuni* were conducted with WGS successfully. However, four isolates cannot be revived, and six failed the WGS. The sequence has been deposited at GenBank under the accession SAMN31577778 to SAMN31577842 and SAMN31577844 to SAMN31577878.

**Figure 1:**
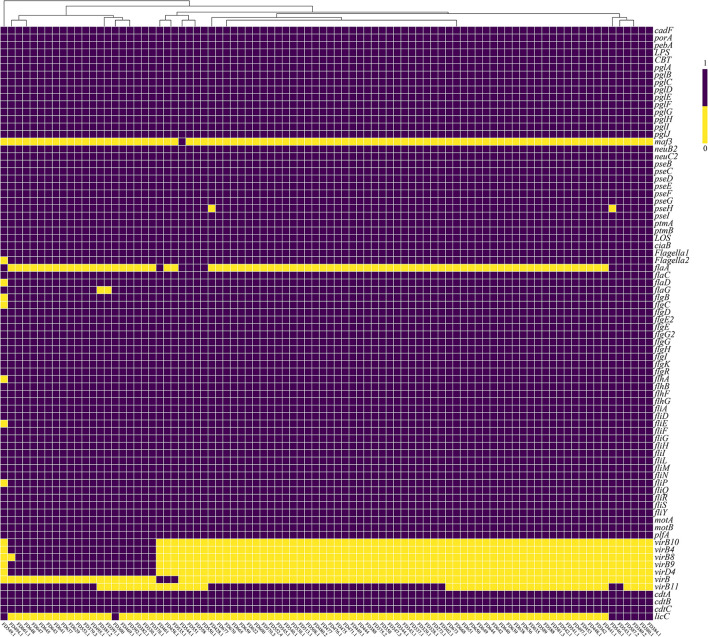
Heatmap of a virulence factor of 88 *C. upsaliensis* isolates from pets in Shenzhen, China. The labels on the X-axis represent the name of the isolates, while labels on the Y-axis correspond to the name of the virulence factor. Value “1” denotes the presence of the virulence factor, whereas value “0” denotes the absence of the virulence factor in the isolates.

According to Figure 1, 100% of *C. upsaliensis* isolates carried the *cadF, porA*, and *pebA* genes, related to adhesion. In addition, all *C. upsaliensis* isolates carried genes associated with N-glycosylation, including the *pglA, pglB, pglC*, and *pglD* genes, and genes related to O-linked flagellar glycosylation, including the *pseB, pseC, pseD*, and *pseE* genes.

Over 97.7% of the *C. upsaliensis* isolates had flagella-related genes, including the *flaC, flaD, flaG, flgB, flgC*, and *flgD* genes, which are involved in motility, chemotaxis, and invasion. However, the *flaA* gene was present in only 13.6% (12/88) of the isolates, while the *flaB* gene was absent.

The *ciaB* gene, which is connected to the invasion, was present in all *C. upsaliensis* isolates. The *ciaC* gene, however, was not found. Moreover, the *cdtA*, c*dtB*, and *cdtC* genes, which code for the cytolethal distending toxin(CDT), were present in all *C. upsaliensis* isolates. Additionally, 21.5% (19/88) of the isolates had the *virB10, virB4, virB8, virB9*, and *virD4* genes related to the type IV secretion system (T4SS), which is involved in secretion and invasion. We present the results in Supplementary Table 2.

### 3.4. Phylogenetic analysis

**Figure 2:**
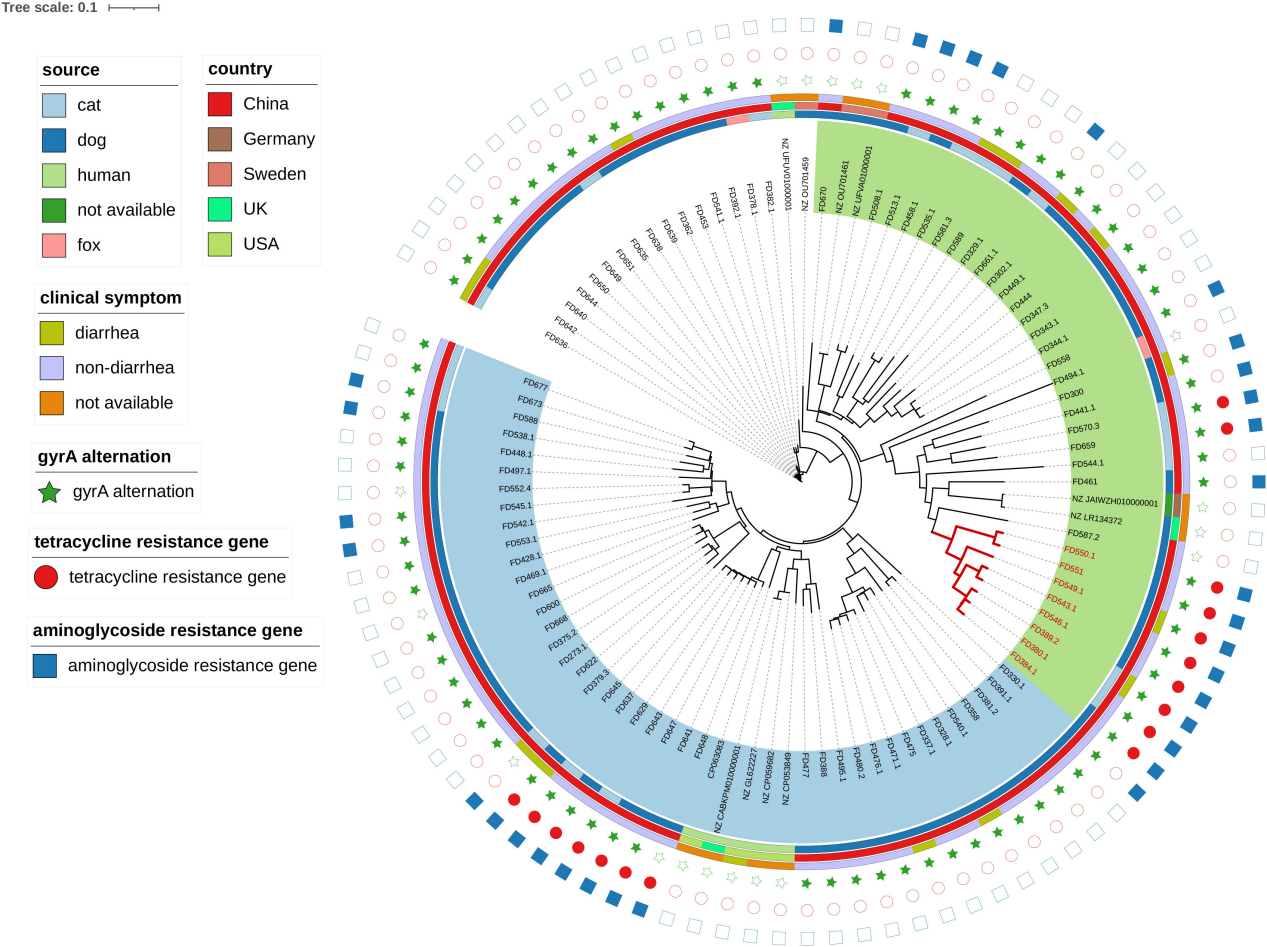
Phylogenetic analysis of 88 *C. upsaliensis* isolates in this study and 11 reference strains by the K-mer tree method. The inner circle represents the source, the middle circle for the country, and the outside circle for the clinical symptom. The stars filled in green represent the *gyrA* gene alteration conferring resistance to fluoroquinolone, the circles filled in red represent the tetracycline resistance gene detected, and the rectangles filled in blue represent the aminoglycoside resistance gene detected. No resistance gene was found, as defined by the shapes without filled color. Two major clades were obtained and labeled with green and blue shades. Red branch isolates were attributed to subclade 1. The tree scale represents a 0.1 change per nucleotide position.

The significant genetic diversity of *C. upsaliensis* isolates in the current study is shown by the phylogenetic analysis in Figure 2. Two major clades were obtained and labeled with green and blue shades. No apparent aggregation was detected in the diarrhea group or the non-diarrhea group. Eight strains in subclade 1 (FD550.1, FD551, FD549.1, FD543.1, FD546.1, FD389.2, FD380.1, and FD384.1) had tetracycline and aminoglycoside resistance genes and the *gyrA* alternation. Moreover, 100% of subclade 1 isolates harbored the *flaA* gene, compared to only 2% (4/80) of non-subclade 1 isolates. In addition, they were phenotypically resistant to six classes of antimicrobials, namely macrolides,/quinolones,/aminoglycosides,/tetracyclines,/lincosamides, and/ketolide.

**Figure 3:**
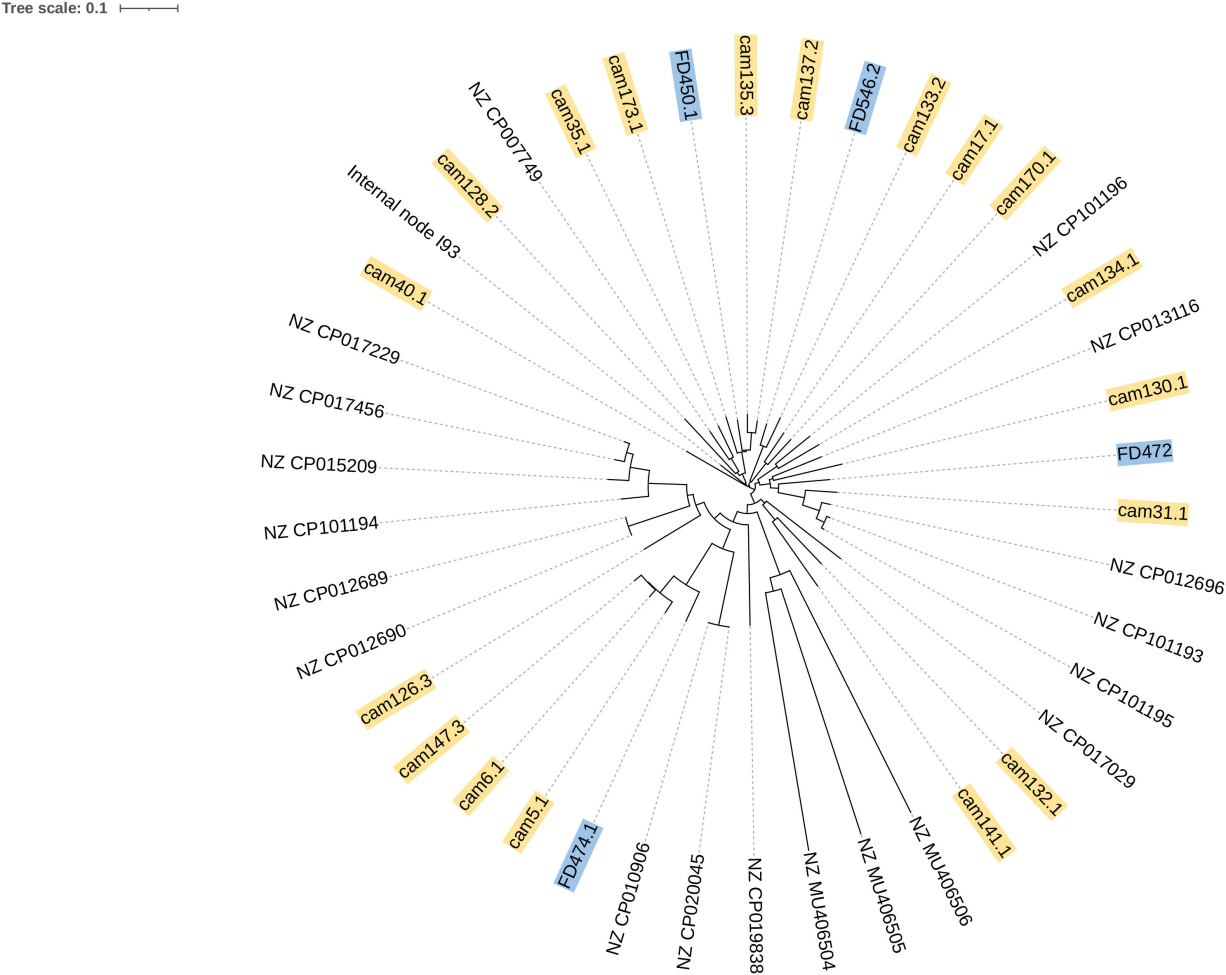
Phylogenetic analysis of four *C. jejuni* isolates in this study and 19 reference strains by the K-mer tree method. The labels in blue were *C. jejuni* isolates in this study, and the labels in yellow were chicken source isolates from 2018 in Shenzhen, China. Other isolates were *C. jejuni* reference strains downloaded from PubMed.

According to the phylogenetic analysis in Figure 3, two *C. jejuni* isolates (FD546.2 and FD450.1) in this study were related to the chicken source *C. jejuni* (cam133.2, cam 135.3, and cam 137.2) from 2018 in Shenzhen, China.

### 3.5. Resistance genotype and concordance with the phenotypic antimicrobial resistance

Blasting with the CARD database found three main genotypical antimicrobial resistance types, namely *gyrA* alternation, aminoglycoside, and tetracycline resistance, consistent with the result of phenotypical antimicrobial resistance. We found the nucleotide mutation C257T in the *gyrA* gene, causing the substitution T86I.

In *C. upsaliensis*, 89.7% (79/88) of the isolates had antibiotic target alteration (C257T) in the *gyrA* gene conferring resistance to fluoroquinolone. In total, 36.4% (32/88) of the isolates carried the aminoglycoside resistance gene and were phenotypically aminoglycoside resistant (gentamycin or streptomycin resistance), comprised of 21 isolates that had the *aph (2*″*)-If* gene, 10 isolates had the *aac (6*′*)-aph (2*″*)* gene, and 1 isolate simultaneously carried the *aph (2*″*)-If* and *aac (6*′*)-aph (2*″*)* genes. However, 20.5% (18/88) of isolates exhibited MIC ≥ 64 μg ml^−1^ against streptomycin while lacking the aminoglycoside resistance gene. In addition, 19.3% (17/88) of the isolates had the tetracycline resistance gene [*tet (O)* or *tet (O/M/O*)] and were phenotypically resistant to tetracycline, whereas 1.1% (1/88) of the isolates were phenotypically tetracycline resistant while lacked tetracycline resistance gene.

All four *C. jejuni* isolates (100%, 4/4) had antibiotic target alteration (C257T) in the *gyrA* gene conferring resistance to fluoroquinolone. In addition, four *C. jejuni* isolates carried the beta-lactam resistance gene; three of them had the *OXA-193* gene, and one had the *OXA-595* gene. In addition, all four isolates had the *cmeR* gene, related to antibiotic efflux pump belonging to resistance-nodulation-cell division (RND) antibiotic, causing resistance to macrolide antimicrobials, fluoroquinolone antimicrobials, cephalosporin, and fusidane antimicrobials. The *cmeR* gene was absent in *C. upsaliensis* and *C. helveticus* isolates in this study. Three *C. jejuni* isolates (75%, 3/4) harbored the tetracycline resistance gene and were phenotypically resistant.

Two *C. helveticus* isolates harbored the aminoglycoside resistance gene [*aph (2*″*)-If* )]and were phenotypically aminoglycoside resistant (gentamycin or streptomycin resistance). Moreover, two *C. helveticus* isolates had the *gyrA* gene alteration (C257T) conferring resistance to fluoroquinolone.

## 4. Discussion

Since dogs and cats are the primary hosts of *C. upsaliensis* and *C. helveticus*, these animals may be crucial in the epidemiology of these species and serve as potential reservoirs for human infection (Acke, 2018). However, little is known about the pet-related *Campylobacter* spp. in China. Our study demonstrated the prevalence and whole-genome sequence profile of *Campylobacter* spp. in pets and indicated the high antimicrobial resistance of the isolates, which was previously unreported in Shenzhen, China.

A meta-analysis research conducted in 2015 from 34 studies on the prevalence of *Campylobacter* spp. in domestic pets found that domestic cats and dogs had a mean prevalence of roughly 25% (Pintar et al., 2015), which is consistent with 33.5% of the pets in this study. Our findings, which agreed with Acke's review (Acke, 2018), showed that *C. upsaliensis* had the highest prevalence (30.2%, 98/325), followed by *C. helveticus* and *C. jejuni*.

Koláčková et al. reported that young dogs on homemade food who have diarrhea might be regarded as a risk group for the potential transfer of *Campylobacter* infections from pets to humans (Koláčková et al., 2015). Karama et al. also reported that age was the only risk factor linked with a higher likelihood of carrying *C. upsaliensis*, and older dogs had a significantly higher prevalence of *Campylobacter* spp. (Karama et al., 2019). In our study, however, no significant difference was found between the diarrhea group and the non-diarrhea group in terms of the prevalence of *Campylobacter* spp. in dogs or cats, younger dogs (3 years old), or older dogs (>3 years old). Additionally, three of the four *C. jejuni* isolates found in this study came from pets that did not have diarrhea. From our study, *Campylobacter* does not necessarily relate to diarrhea in pets.

Our study also demonstrated that *Campylobacter* spp. from pets exhibited similar high resistance to antimicrobials as isolates from the clinical patient. The high antimicrobial resistance rate of *Campylobacter* is an increasing problem in public health (Contreras-Omaña et al., 2021; Rahman et al., 2021; Wallace et al., 2021; Eryildiz et al., 2022; Sasaki et al., 2022). In this study, MDR was found in 55.1% of the *Campylobacter* isolates. The main MDR pattern was aminoglycosides/quinolones/lincosamides, which were comparable with the antimicrobial resistance profile of isolates from clinical patients (Wieczorek et al., 2018; Frazão et al., 2021; Mencía-Gutiérrez et al., 2021).

Moreover, our findings confirmed the variety of virulence factors that *Campylobacter* spp. carried. Virulence factors can be divided into bacterial chemotaxis, motility, attachment, invasion, survival, cellular transmigration, and spread to deeper tissue (Tegtmeyer et al., 2021). The primary flagellar genes associated with strain motility are the *flaA* and *flaB* genes (Lopes et al., 2021). Only 13.6% (12/88) of the isolates in this study had the *flaA* gene, and no isolate had the *flaB* gene. We used the *Campylobacter* isolation kit incorporating a membrane filter method, and only dynamic isolates can penetrate the filtration membrane. Based on the experimental results, we confirmed that the *flaB* gene is not essential for total motility in *Campylobacter*, which was reported previously (Koolman et al., 2015).

The most interesting finding was that subclade 1 contained eight isolates that displayed phenotypically resistance to six classes of antimicrobials and had resistance genes against tetracyclines and aminoglycosides and harbored the *gyrA* gene alteration. Additionally, all isolates in subclade 1 possessed the *flaA* gene, which was uncommon outside subclade 1, suggesting isolates in subclade 1 may have more advantage in mobility.

Goyal et al. demonstrated an outbreak of pet store puppy-associated extensively drug-resistant *C. jejuni* infections (Goyal et al., 2021). In addition, Thépault et al. reported that genotype comparison with previously characterized isolates revealed a partial overlap among *C. jejuni* isolates from pets, chickens, cattle, and clinical cases (Thépault et al., 2020). This overlap suggests the potential role of livestock and humans in pets' exposure to *Campylobacter* or vice versa.

Furthermore, concordance between the genotypically and phenotypically resistance against antimicrobials was observed in *C. upsaliensis*. The percentages of phenotypically resistant isolates against ciprofloxacin, gentamicin, and tetracycline were 94.9%, 43.9%, and 21.4%, respectively. Accordingly, the percentages of genotypically resistant isolates against fluoroquinolone, aminoglycosides, and tetracyclines were 89.7%, 36.4%, and 19.3%, respectively. Interestingly, 18 *C. upsaliensis* isolates showed high MICs (≥ 64 μg ml^−1^) against streptomycin without an aminoglycoside resistance gene detected. We referred that this aminoglycoside resistance is not conferred by known resistance genes. In addition, concordance between the genotype and phenotype was influenced by the computational pipelines, genome coverage, and the type of ARG but not by input data (Hodges et al., 2021).

Our study had limitations, including a lack of adequate geographic representation, a short sampling season, and a failure to isolate from the patient and pet simultaneously. These shortcomings led to an inadequate impact of the epidemiological analysis, which should be improved in future research.

Our analysis concluded that pets are a significant source of *Campylobacter* spp. strains and a potential reservoir for them. In particular, *C. upsaliensis* of subclade 1 in this study required additional investigation because of its broad MDR phenotype and relatively high prevalence of the *flaA* gene.

## Data availability statement

The data presented in the study are deposited in the NCBI repository, BioProject accession number PRJNA897092.

## Ethics statement

The animal study was reviewed and approved by the Institutional Review Board of the Nanshan Center for Disease Control and Prevention. Written informed consent was obtained from the owners for the participation of their animals in this study.

## Author contributions

YD and MZ designed the experiments. CJ, YM, BZ, MY, and JH participated in the sample collection and performed the experiments. GZ and HW carried out the genome bioinformatics study. CJ and YM wrote the manuscript. The submitted manuscript was read and approved by all authors.
