# Erratum: Prevalence, genomic characterization and antimicrobial resistance of *Campylobacter* spp. isolates in pets in Shenzhen, China

**DOI:** 10.3389/fmicb.2023.1245222

**Published:** 2023-07-04

**Authors:** S Frontiers Production Office

**Affiliations:** Frontiers Media SA, Lausanne, Switzerland

**Keywords:** *Campylobacter upsaliensis*, *Campylobacter jejuni*, *Campylobacter helveticus*, whole-genome sequencing, antimicrobial resistance, matrix-assisted laser desorption ionization-time of flight mass spectrum, pets, virulence-associated genes

Due to a production error, the authors “Changyan Ju” and “Yanping Ma” sharing first authorship was not indicated clearly with the “dagger mark”.

The correct statement to follow is “These authors share first authorship”.

The publisher apologizes for this mistake. The original article has been updated.

